# Comparative satisfaction of receiving medical abortion service from nurses and auxiliary nurse-midwives or doctors in Nepal: results of a randomized trial

**DOI:** 10.1186/s12978-017-0438-7

**Published:** 2017-12-16

**Authors:** Anand Tamang, Iqbal H. Shah, Pragya Shrestha, I. K. Warriner, Duolao Wang, Kusum Thapa, N. T. My Huong, Olav Meirik

**Affiliations:** 1 Center for Research on Environment Health and Population Activities (CREHPA), Kusunti, Lalitpur, P.O. Box 9626, Kathmandu, Nepal; 2 Department of Global Health and Population, Harvard T. H. Chan School of Public Health, Boston, USA; 3 School of Education, University of Delaware, Newark, Delaware USA; 4 Chicago, USA; 5 Liverpool School of Tropical Medicine, Liverpool, UK; 6 Nepal Society of Obstetricians and Gynaecologists (NESOG), Kathmandu, Nepal; 7 UNDP/UNFPA/UNICEF/WHO/World Bank Special Programme of Research, Development and Research Training in Human Reproduction (HRP), Department of Reproductive Health and Research (RHR), World Health Organization, Geneva, Switzerland; 8 Instituto Chileno de Medicina Reproductive (ICMER), Santiago, Chile

**Keywords:** Medical abortion, Nepal, Nurses, Auxiliary nurse-midwives, Doctors, Satisfaction

## Abstract

**Background:**

Early first-trimester medical abortion (MA) service (≤ 63 days) has been provided by doctors and nurses under doctors’ supervision since 2009 in Nepal. This paper assesses whether MA services provided by specifically trained and certified nurses and auxiliary nurse-midwives independently from doctors’ supervision, is considered as satisfactory by women as those provided by doctors.

**Methods:**

The data come from a multi-center, randomized, controlled equivalence trial conducted between April 2009 and March 2010 in five district hospitals in Nepal. Women seeking MA were randomly assigned to doctors or nurses and auxiliary nurse-midwives(ANMs).Eligible women were administered 200 mg mifepristone orally followed by 800 μg misoprostol vaginally two days later by their assigned providers and followed up 10–14 days later. At the follow-up visit women’s reported satisfaction with MA service they received was measured.

**Results:**

Of 1295 women screened for eligibility, 535 were randomly assigned to a doctor and 542 to a nurse or ANM. Nineteen women were lost-to-follow up in the former group and 27 were lost-to-follow up or did not complete the acceptability interview in the latter group. This study is, therefore, based on516womenin the doctor’s group and 515 women in the nurse or ANM group. All women in the nurse or ANM group reported being satisfied or highly satisfied by MA compared to 99% in the doctor’s group. Satisfaction was similar regardless of the type of provider; 38% among nurse or ANM and 35% among the doctor group were “highly satisfied”, and 62% and 64%, respectively, were “satisfied”. Women’s experiences such as ‘less than expected amount or duration of bleeding following MA’, ‘shorter than expected duration of the abortion process’, and ‘able to manage symptoms’, were found to be associated with women’s higher satisfaction with MA. Counseling and information on the method, potential complications of MA and post-abortion contraception was nearly universal. No statistically significant differences were found in the level of satisfaction by age, parity, marital status, education or occupation of women.

**Conclusions:**

Women’s satisfaction with MA service provided by trained nurses or auxiliary nurse-midwives was similar to that provided by doctors. The findings, therefore, provide support for extending safe and accessible medical abortion services by government-trained nurses and auxiliary nurse midwives to women seeking early first trimester pregnancy termination.

**Trial registration:**

The trial was retrospectively registered with ClinicalTrials.gov (identifier: NCT01186302). Registered August 20, 2010.

## Plain English summary

This paper examines whether the satisfaction and preference of women differ if they receive medical abortion service from nurses or auxiliary nurse-midwives as compared to doctors in district hospitals in rural Nepal. Women requesting abortion within the first 63 days of pregnancy were assigned by chance either to a group of trained nurses or auxiliary nurse-midwives or to a group of doctors. The social and background characteristics of women in the two groups were broadly similar. Women were satisfied with the medical abortion service they received, irrespective of who provided it – nurses, auxiliary nurse-midwives or doctors. The authors conclude that women seeking early medical abortion are equally satisfied by the medical abortion services they received from a trained nurse and nurse-midwife or by a doctor. This finding is important in the resource-poor setting of Nepal where majority of population live in rural areas and doctors are few. In such settings, government-trained nurse and nurse-midwife can provide medical abortion service which women consider as satisfactory as the service provided by doctors.

## Background

Medical abortion (MA) is safe and effective non-surgical option for women who wish to end a pregnancy in the early part of the first-trimester but do not want to undergo surgical methods of vacuum aspiration or dilation and curettage [1]. MA has been shown to be widely acceptable to women in both developed and developing countries [2–5]. Although, different MA drugs have been used, alone or in combination, to induce abortion, a combined regimen of mifepristone and misoprostol is most widely used. The World Health Organization (WHO) recommends an initial dose of mifepristone followed by misoprostol 24 to 48 h later for early first trimester abortion (up to 9 weeks of gestation). Efficacy rates up to 98% are reported using this regimen [6].

When given the choice, many women prefer MA to the surgical alternative. Researchers in Nepal interviewed 499 women who chose MA and 530 women who chose manual vacuum aspiration and found that the odds of choosing MA were more than three times higher among women who knew about both methods (60% choosing MA)compared to those who did not (30% choosing MA). Of those who had decided on MVA prior to receiving information at the clinic, 29% chose MA. In contrast, only 10% of those who had intended to have MA opted for MVA after receiving information and counseling. Women who had higher education or resided in urban areas were more likely to choose MA [7]. A study in Mexico City showed that not only the safety and effectiveness of MA by physicians and nurses were similar but both type of providers were equally and highly acceptable to women with 98% indicating that they would recommend the type of provider they received the service from to a friend if she needed the same procedure [8]. Among women receiving MA service from nurses, 79% were ‘very satisfied’ with the provider as compared to 76% in the group of doctors.

In Nepal, the provision of MA using the combined regimen of mifepristone and misoprostol began in 2009. Initially, doctors as well as nurses under doctors’ supervision were permitted to provide MA for less than nine completed weeks of pregnancy. The government of Nepal is keen on expanding MA services in rural areas by training non-physician clinicians with a particular focus on nurses and auxiliary nurse-midwives (ANMs) at primary healthcare centers and seeking evidence to support that such service would be safe and effective as well as acceptable to women.

MA has enormous potential to increase access to safe abortion. The provision of MA by nurses and ANMs has proven to be safe, effective and acceptable [4–6]. However, evidence remains scant in Nepal on whether women are equally satisfied with MA provision by nurses or ANMs as by doctors. Nurses and ANMs are closest to women geographically and socially. Yet women may doubt their skills in providing safe MA services compared to doctors. This study, therefore, seeks to provide evidence on women’s reported satisfaction with MA service provided by nurses or ANMs compared to doctors.

## Methods

### Study aim, design, and setting

This study was part of a multicenter, randomized, controlled equivalence trial to assess outcomes of medical abortion services provided by nurses and ANMs and doctors in Nepal [1]. Women seeking termination of early first-trimester pregnancy were randomly assigned to MA managed by doctors or by trained nurses or ANMs and followed up for completeness of abortion and any complications. Data were collected between April 15, 2009 and March 17, 2010 in five district hospitals in hilly regions (Bhaktapur, Baglung, Dhading) and lowland regions (Rupendehi, Chitwan) in Nepal. Participating hospitals had sufficient staff to establish a group of doctors and a group of nurses and ANMs for implementation of the study. To ensure independent provision of services and to reduce interactions between the two groups, each study site established separate waiting areas and examination rooms for nurses and ANMs and for doctors.

### Characteristics of participants

Women met medical eligibility criteria if their pregnancy was less than 9 weeks (≤63 days) as estimated by date of last menstrual period and by abdominal and bimanual pelvic examination. Women were eligible if they were older than 16 years, resided no more than 90 min journey from the study clinic, and were willing to be randomly assigned to a doctor or to a nurse and ANM, to return to the clinic 10–14 days after the start of treatment, and to provide written informed consent.

A woman was ineligible if she had any contraindication to MA, previous allergic reaction to any of the drugs in the MA regimen, known or suspected ectopic pregnancy or undiagnosed adnexal mass, inherited porphyria, chronic adrenal failure, long-term corticosteroid therapy, hemorrhagic disorder or anticoagulant therapy, or an intrauterine device that could not be removed before administration of mifepristone.

### Procedures

Eligible women were randomly assigned either to a doctor or to a nurse or ANM using a computer-generated randomization scheme classified by the participating center with a block size of six. The random allocation was indicated in sealed opaque envelopes, numbered consecutively. Women knew that they could be either in the doctors’ or nurses or ANMs’ group and that both groups provided MA service from the same facility. Following the written informed consent, a research assistant opened the envelope and assigned women to the allocated group. Nurses and ANMs consisted of eight Staff Nurses and three ANMs. To be a staff nurse, one needs to complete a three years’ Proficiency Certificate Level nursing course after completing 10th grade. Whereas, ANM follows an 18-month course after completing 10^th^grade. The doctors were six obstetricians and gynaecologists, three general practitioners, and five doctors with a Bachelor of Medicine and Bachelor of Surgery Degree. Participating providers underwent a 3-day training course in MA and were certified by the National Health Training Centre.

### Study procedures

Clinical procedures for the medical abortion followed the Nepalese MA protocol at the time of the study. The regimen consisted of one 200 mg tablet of mifepristone and four 200 μg tablets of misoprostol packaged in a single blister pack (Medabon, Sun Pharmaceutical Industries Ltd., Mumbai, India). The assigned provider gave the woman the mifepristone tablet orally on day 1 and administered the misoprostol tablets vaginally on day 3. After misoprostol insertion, the woman was monitored in hospital for three hours. Her assigned provider examined her bimanually to assess the status of the abortion before she left the hospital. Ten to 14 days after the administration of mifepristone the woman returned for a follow-up visit to the same assigned provider for a clinical assessment of the outcome of the treatment. Additional follow-up visits were scheduled with the assigned provider if needed, and women were encouraged to return at any time for unscheduled visits. In addition to the clinical assessment, an “Acceptability Form” was administered by the female research assistants to ascertain women’s experiences of symptoms and their satisfaction with MA. Women were asked about their experiences/perceptions regarding pain, amount of bleeding, duration of abortion process, side effects and privacy. For each of these five conditions, women were asked to respond either ‘less than expected’, ‘same as expected’, ‘more than expected’, or ‘no comment’. Questions measuring the coverage and quality of counseling and communication included: (1) “was the method explained clearly to you before taking tablets”; (2) “were the signs of complications explained clearly to you”; (3) “did you receive counseling on contraceptive use”; and (4) “did the provider give you an opportunity to ask questions”. Preferences of sex of the provider and the length of practice were also ascertained. In addition, all women were asked one direct question on ‘satisfaction’: “how satisfied are you with the method”.

The study defined, a priori, serious adverse event to include: i) hemorrhage (excessive bleeding as measured by either over 500 ml equivalent to soaking 1 pad per hour for longer than 10–12 h or local equivalent) that requires a blood transfusion, and ii) infection that requires hospitalization. Pelvic infection subsequent to medical abortion is usually the result of retention of incomplete abortion or genital tract infection. Symptoms may range from mild to severe; they include chills, fever, sweats, abnormal-smelling vaginal discharge, abdominal pain or cramps, uterine and cervical motion tenderness at pelvic exam, rebound tenderness, distended abdomen, mildly low blood pressure, prolonged bleeding, overall malaise, and purulent cervical discharge. Adverse events were defined to include such side-effects of MA drugs such as nausea, vomiting, diarrhea, mild fever, etc.

Female research assistants with backgrounds in nursing, health, or social sciences screened women for eligibility and interviewed them for data collection throughout the study. Providers reviewed the forms completed by the research assistants including the Acceptability Form and had full responsibility for clinical management of each case. They made the final decision on the eligibility of a woman for MA and to the study after physical examination, and recorded clinical data on physical examinations, administration of the drug, case management, and abortion outcome. The study made provisions within the study sites for the management of any adverse event or serious adverse events resulting from the MA drug.

### Conceptual framework

**Fig. 1:**
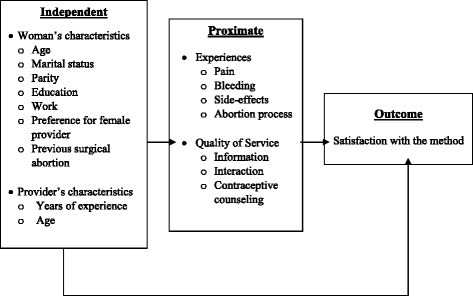
Proximate determinates of satisfaction with medical abortion method

Ascertaining client or user satisfaction with the method and/or the service has been a key to measuring quality of family planning programs and MA services [9–18]. More recently, client satisfaction has been shown as the human rights-related outcome for human rights standards in relation to family planning program implementation [19]. Measuring “satisfaction” is, however, neither straightforward nor easy to interpret [20]. “Satisfaction” can be measured subjectively by one or more direct question(s) to respondents or experiences of care through a series of questions such as the waiting time to receive the service; client-provider interaction and communication [20]. Personal characteristics of the provider and recipients of service also play a role in satisfaction reported by the latter. It is challenging, therefore, to develop a conceptual framework that encompasses these varied dimensions and guide the selection of variables and the analysis of factors associated with women’s reported satisfaction of medical abortion service received from doctors or nurse and ANM. In this study, we analyze “satisfaction” with response categories: “highly satisfied”; “satisfied”; “not satisfied”; or “no opinion”. This provides an overall subjective assessment of a woman with no indications of underlying factors. In order to address these factors, we adapted the “proximate determinants” framework developed by John Bongaarts [21] originally to explain the variations in fertility levels across countries. The proximate determinants of reported satisfaction with MA (the ‘outcome’ variable in this study) include women’s experiences of pain, excessive bleeding, side-effects and the time it took to complete the abortion from induction of labor. The coverage and quality of counseling and communication measures included in this study are: information provided on the method prior to taking MA tablets; explanation of signs of complications; opportunity provided to ask questions and whether contraceptive counseling was provided. Women with successful abortion outcome, that too within a short or expected duration; experience of less or as expected amount of bleeding and pain not lasting for more than the duration informed by the health provider during counseling, are hypothesized to express satisfaction with the MA service. Better quality counseling is hypothesized to be associated with higher level of satisfaction with the MA service. Both the outcome and the proximate determinants are influenced by the independent background characteristics of women and providers. The background (independent) characteristics of women include age, marital status, parity, education, occupation, previous induced surgical abortion, and preference for female provider. These may influence women’s satisfaction with MA directly or through the proximate determinants. The providers’ characteristics considered in the analysis include type of provider (doctor or nurse and ANM), age, and years of experience in providing abortion services. The framework (Fig. 1) guided the selection of variables and analysis of data.

### Statistical analysis

Women who had completed the “Acceptability Form” during the follow-up visits (10–14 days after abortion treatment) were included in the satisfaction analysis corresponding to 95% of all women randomly assigned to the group of nurse or ANM and 96% of those to the group of doctor. The analyses of women’s satisfaction were performed based on intention to treat principle. Percentages were calculated for women’s responses on their level of satisfaction with MA for both providers’ groups. Among 1031 women, two had “no opinion” and an additional two indicated being “not satisfied” while the rest (1027 women) were either “highly satisfied” or “satisfied”. Logistic regression was applied to identify the factors associated with being “highly satisfied” with MA compared to “satisfied” for 1027 women. Results presented here are based on the best fit model. The analyses were performed using Statistical Package for Social Sciences (SPSS) version 13. Statistical significance was defined for *P* values less than 0.05.

## Results

Of 1295 women initially screened for eligibility, 190 were not eligible for medical, geographical, or follow-up scheduling reasons and one woman declined to take part in the study. The remaining 1104 women were randomized: 552 allocated to nurse and ANM group and 552 to doctor group. After clinical examination, in the nurses or auxiliary nurse-midwives group, 10 women were excluded: in eight, the duration of gestation was longer than 9 weeks; one had signs of ectopic or adnexal mass; and one was diagnosed with cervical polyps. In the doctors’ group, 15 women were excluded: in 12, the duration of gestation was longer than 9 weeks; one had signs of ectopic or adnexal mass; one was not pregnant; and one had a miscarriage before receiving any drugs. Additionally, two women withdrew from the study before physical examination when they were told of their group allocation.

In the nurses and ANMs group, 24 of 542 (4%) women receiving MA were lost to follow-up and three additional women did not complete the acceptability interview. In the doctors’ group, the loss to follow-up was 19 of 535 (4%) women. In both groups, women lost to follow-up were, on average, less educated than those followed up. Therefore, 515 women in the nurses or ANMs group and 516 in the doctors’ group were included in the satisfaction analysis reported in this paper.

The mean age of doctors was 42.4 years (standard deviation or SD = 11.0) while that of the nurses or ANMs was 41.7 years (SD = 7.1). All the nurses or ANMs were female. Among the doctors, 79% were male. The average number of years of professional medical practice for nurses or ANMs was 21.8 years as compared to 14.1 years for doctors.

The participating women in both groups had similar background characteristics (Table 1). In general, nearly all women were married or cohabitating and their average age was 28 years. Almost three-fourths of women were house-wives and nearly one-third of them had never been to school or did not complete primary education. In both provider groups, 30% of women seeking MA have given three or more births. Nearly two-third of them had no previous surgical abortion.

**Table 1:** Characteristics of women undergoing early medical abortion

	Nurses and auxiliary nurse-midwives (*n* = 515)	Doctors (*n* = 516)	Total (*n* = 1031)
Age			
Mean (SD)	27.9(6.0)	28.0 (5.7)	28.0(5.8)
< 20	30 (5.8%)	27 (5.2%)	57 (5.5%)
20–29	284 (55.1%)	300 (58.1%)	584 (56.6%)
30–39	177 (34.4%)	165 (32.0%)	342 (33.2%)
40+	24 (4.7%)	24 (4.7%)	48 (4.7%)
Marital status			
Married or cohabiting	503 (97.7%)	509 (98.6%)	1012 (98.2%)
Separated/divorced/widowed	3 (0.6%)	1 (0.2%)	4 (0.4%)
Single	9 (1.7%)	6 (1.2%)	15 (1.5%)
Education			
Never attended/primary incomplete	155 (30.1%)	145 (28.1%)	300 (29.1%)
Primary school	97 (18.8%)	83 (16.1%)	180 (17.5%)
Lower secondary school	75 (14.6%)	68 (13.2%)	143 (13.9%)
Secondary school	111 (21.6%)	134 (26.0%)	245 (23.8%)
More than secondary school	77 (15.0%)	86 (16.7%)	163 (15.8%)
Occupation			
Student	34 (6.6%)	31 (6.0%)	65 (6.3%)
Housewife	380 (73.8%)	374 (72.5%)	754 (73.1%)
Manual laborer/farmer	65 (12.6%)	49 (9.5%)	114 (11.1%)
Professional	33 (6.4%)	59 (11.4%)	92 (8.9%)
Others	3 (0.6%)	3 (0.6%)	6 (0.6%)
Live births			
Mean (SD)	2.4 (1.2)	2.3 (1.1)	2.3 (1.2)
No data	49 (9.5%)	46 (8.9%)	95 (9.2%)
0	2 (0.4%)	3 (0.6%)	5 (0.5%)
1–2	311 (60.4%)	312 (60.5%)	623 (60.4%)
3+	153 (29.7%)	155 (30.0%)	308 (29.9%)
Previous induced surgical abortions^a^			
Mean (SD)	0.4 (0.8)	0.4 (0.7)	0.4 (0.8)
No data	49 (9.5%)	46 (8.9%)	95 (9.2%)
0	345 (67.0%)	325 (63.0%)	670 (65.0%)
1	82 (15.9%)	107 (20.7%)	189 (18.3%)
2+	39 (7.6%)	38 (7.4%)	77 (7.5%)
Gestational age			
Mean (SD)	6.9 (1.01)	6.6 (1.0)	6.8 (1.01)
5	23 (4.5)	35 (6.8)	58 (5.6)
6	202 (39.2)	271 (52.5)	473 (45.9)
7	125 (24.3)	72 (14.0)	197 (19.1)
8	141 (27.4)	125 (24.2)	266 (25.8)
9	24 (4.7)	13 (2.5)	37 (3.6)

^a^Medical abortion (*MA*) was introduced in Nepal in 2009 just few months ahead of the study and only 18 women participating in this study had previously used MA. It is was not asked where they obtained the MA from and the abortion outcome with MA use, hence these conditions were excluded

Complete abortion rates were 97.3% for nurses or ANMs’ groupand 96.1% for doctors’ group, following intention-to-treat approach. The risk difference (95% confidence interval or CI) for complete abortion was 1.24% (−0.53%, 3.02%), which falls within the pre-defined equivalence range (−5%, 5%). There were 5 cases (1%) of failed abortion in the doctor arm and none in the nurse and ANM group; the remaining cases were incomplete abortions. There were no serious complications and no difference in side effects by type of provider. Nurses or ANMs reported discussing less than 2% of cases with other providers and referred 1% of cases to other providers, a pattern similar to that of doctors.

Table 2 shows women’s subjective assessment of MA experience as well as their perceptions of privacy of service by type of provider. Overall, differences by type of provider were negligible. In the nurses and ANMs’ group, 27.8% of women and in doctors’ group 26.9% reported that the amount of bleeding was “more than expected”. The duration of the abortion process and other side effects such as diarrhea, vomiting, nausea, chills and fever reported as “more than expected” by the participating women was below 4% for both types of providers. “More than expected” pain was reported slightly more by women in doctors’ group (21.5%) compared to nurses and ANMs’ group (16.5%).Less than 2% of women in each group reported of privacy being “less than expected”.

**Table 2:** Experience of medical abortion procedure

	Nurses or auxiliary nurse-midwives (*n* = 515)				Doctors (*n* = 516)			
Symptoms	Less than expected	Same as expected	More than expected	Cannot comment	Less than expected	Same as expected	More than expected	Cannot comment
Pain	135 (26.2)	288 (55.9)	85 (16.5)	7 (1.4)	138 (26.7)	262 (50.8)	111 (21.5)	5 (1.0)
Amount of bleeding	45 (8.7)	327 (63.5)	143 (27.8)	0 (0.0)	39 (7.6)	333 (64.5)	139 (26.9)	5 (1.0)
Duration of abortion process	32 (6.2)	404 (78.4)	18 (3.5)	61 (11.8)	27 (5.2)	409 (79.3)	17 (3.3)	63 (12.2)
Privacy	6 (1.2)	353 (68.5)	152 (29.5)	4 (0.8)	9 (1.7)	345 (66.9)	158 (30.6)	4 (0.8)
Other side-effects	181 (35.1)	174 (33.8)	3 (0.6)	157 (30.5)	202 (39.1)	164 (31.8)	1 (0.2)	149 (28.9)

The quality of counseling proximate determinants were similar in the two providers groups. Women reported nearly identical levels of satisfaction (Table 3). Before taking medical abortion tablets, all but one woman in the doctor group reported that they had been clearly explained the method, the signs of complications and received counseling on contraceptive use. Three women in nurses or ANMs’ group and four in the doctors’ group reported that the provider did not give them an opportunity to ask questions.

**Table 3:** Quality of services indicators

	Nurses or auxiliary nurse-midwives (*n* = 515)	Doctors (*n* = 516)
Was the method explained clearly to you before taking tablets?		
No	0 (0)	1 (0.2%)
Yes	515 (100%)	515 (99.8%)
Were the signs of complications explained clearly to you?		
No	0 (0)	1 (0.2%)
Yes	515 (100%)	515 (99.8%)
Do you receive counseling on contraceptive use?		
No	0 (0)	1 (0.2%)
Yes	515 (100%)	515 (99.8%)
Did the provider give you the opportunity to ask questions?		
No	3 (0.6%)	4 (0.8%)
Yes	512 (99.4%)	512( 99.2%)
Please tell me how important it is to you that the provider has many years of experience?		
Very important	487 (94.6%)	493 (95.5%)
Somewhat important	27 (5.2%)	22 (4.3%)
Not very important	1 (0.2%)	1 (0.2%)

Overall, 99.6% of the participating women reported to be “satisfied” or “highly satisfied” with MA (Table 4). There was little difference in women’s satisfaction level by the type of provider although the number of women who reported “highly satisfied” was slightly more in the nurses or ANMs’ group than in the doctors’ group. A negligible percentage (0.4%) of women in the doctors’ group reported being “not satisfied” or had “no opinion”.

**Table 4:** Satisfaction of medical abortion procedure by type of provider

Level of satisfaction	Nurses or auxiliary nurse-midwives (*N* = 515)	Doctors (*N* = 516)	Total (*N* = 1031)
Highly satisfied	198 (38.4)	180 (34.9)	378 (36.7)
Satisfied	317 (61.6)	332 (64.3)	649 (62.9)
Not satisfied	0 (0.0)	2 (0.4)	2 (0.2)
No opinion	0 (0.0)	2 (0.4)	2 (0.2)

To examine the relative role of proximate determinants and of independent variables shown in Fig. 1, controlling for their confounding effects, we applied logistic regression. Results (Table 5) indicate that the odds of being highly satisfied for women with higher gestational age (8–9 weeks) were 1.4 times (95% CI 1.1–1.9) than that for women with shorter (5–7 weeks) gestational age. Unsurprisingly, women who experienced a shorter abortion process were more likely to be highly satisfied. The odds ratio (OR) of being highly satisfied for women whose bleeding was less than they expected were 1.5 (95% CI 1.1–2.2) compared with their counterparts. Women who reported a shorter than expected duration of abortion process had 1.5 times (95% CI 0.6–3.7) odds to be highly satisfied. The odds of being highly satisfied for women with “shorter than expected or less” duration of bleeding were almost four times (95% CI 1.4–9.8) compared to their counterparts.

**Table 5:** Factors associated with “high satisfaction” of medical abortion

	Percent	Number	OR	95% C.I.
Age				
< 25	35.9	306	1.00	
25–34	36.2	550	0.98	0.68–1.40
35+	40.4	171	1.19	0.74–1.90
Marital status				
Married or cohabiting	37.1	1008	1.00	
Separated/divorced/widowed/single	21.1	19	0.46	0.14–1.58
Education				
Never attended/primary incomplete	37.8	299	1.00	
Primary to Lower secondary	36.1	321	1.06	0.73–1.54
Secondary or more	36.6	407	0.99	0.68–1.46
Occupation				
Housewife	35.9	750	1.00	
Manual laborer/farmer	36.8	114	0.87	0.55–1.38
Professional	44.6	92	1.23	0.74–2.03
Student/Others	36.6	71	1.04	0.51–2.13
Live births				
Nulliparae	35.0	100	1.00	
Multiparae	37.0	927	0.89	0.46–1.71
Previous induced surgical abortion				
No	36.3	763	1.00	
Yes	38.3	264	1.05	0.75–1.45
Gestational age	*			
5–7 weeks	34.8	725	1.00	
8–9 weeks	41.7	302	1.43^*^	1.06-1.92
More than expected pain				
Yes	33.5	194	1.00	
No	37.6	833	1.14	0.79–1.65
More than expected amount of bleeding	**			
Yes	29.3	280	1.00	
No	39.6	747	1.54^*^	1.06-2.23
Longer than expected duration of abortion process	*			
Yes	20.6	34	1.00	
No	37.4	993	1.52*	0.62–3.73
Duration of bleeding	***			
Too long	20.7	58	1.00	
Long but acceptable	34.2	243	1.22	0.58–2.57
As expected	37.1	677	1.56	0.75–3.28
Shorter than expected or less	65.3	49	3.73^**^	1.42–9.77
Treatment of symptoms by women	***			
No	22.0	209	1.00	
Yes	40.6	818	2.00^***^	1.34-2.98
Type of Provider				
Doctors	35.2	512	1.00	
Nurses/ANMs	38.4	515	0.92	0.67–1.26
Provider’s age	***			
24–34	14.7	190	1.00	
35–44	37.1	256	3.97^***^	2.29-6.90
45+	43.9	581	5.06^***^	3.00-8.54
Experience of abortion services of provider				
< 2 years	33.5	319	1.00	
2+	38.3	708	0.74	0.52–1.07
Preference for female provider	***			
Yes	30.1	657	1.00	
No	48.6	370	1.70^***^	1.28-2.27
Total	36.8	1027	0.03	
-2 Log likelihood			1211.68	
Cox & Snell R Square			0.13	
Nagelkerke R Square			0.17	

^***^Significant at *P* ≤ 0.001, ^**^significant at *P* ≤ 0.01, and ^*^significant at *P* ≤ 0.05

The provider’s age was also statistically significant with higher age positively associated with women reporting highly satisfied. Women whose service providers were aged 45 and above had 5 times odds (95% CI 3.00–8.5) of being highly satisfied as compared to their counterparts, while those served by providers aged between 35 years and 44 years had almost 4 times (95% CI 2.3–6.9) odds compared with women who were provided MA service by providers of younger age. Provider type was found to be insignificant in both simple and multivariate analyses. Therefore, satisfaction of MA among women in Nepal was found to be independent of the type of provider.

Most women in both the doctor and nurse and ANMs groups reported that the MA had no event or symptom associated with major discomfort (results not shown here). For women in the doctors’ group the most frequently reported worst feature of the MA process was the discomfort of vaginal examination(14%) and the second common worst feature was bleeding (9%), whereas for women in the nurse and ANMs group the most reported worst feature was bleeding (11%) followed by the discomfort of vaginal examination (7%).

Results on safety and effectiveness of MA [1] showed that 11 women in the nurse or auxiliary nurse-midwives group and 18 women in doctors’ group had incomplete and continuing pregnancies which were terminated by manual vacuum aspiration by their respective providers. All of these women reported satisfaction with the procedure, three of whom reported being highly satisfied with the abortion (two women in the nurse and auxiliary nurse-midwives group and one in the doctor group).No serious adverse events were recorded. Women reported typical side-effects such as nausea, vomiting, diarrhea, abdominal pain, chills, and fever with no difference by type of provider.

## Discussion

Results on the outcome of effectiveness and safety of the randomized controlled equivalence trial are reported elsewhere [1].This paper focused on women’s reported satisfaction of MA services provided by nurses or ANMs as compared to doctors. In this study, we define satisfaction with MA in terms of women’s experiences of pain, the amount and duration of bleeding and side effects associated with MA and through an overall subjective assessment of satisfaction with the procedure. Satisfaction with MA was reported by almost all participating women. Thirty-eight percent of women in the nurses or ANMs group and 34.9% in the doctors’ group reported being “highly satisfied”. The regression results confirm that satisfaction of MA among women is independent of the provider being a doctor or a nurse or ANM.

Privacy has been noted as one of the most valued features among women seeking abortion. Our study indicates that the majority of the women considered privacy to be the same or better than they had expected and was not associated with the type of provider. Less than 2% of women in the nurse or ANMs’ group and in doctors’ group, reported privacy to be less than expected. However, one should note that the study was conducted in district hospitals where separate rooms were available to ensure privacy. At lower level health facilities such as sub health posts, privacy by provider type may differ with no or little privacy for women seeing nurses and ANMs than doctors. In Nepal, lower level health facilities are often crowded and privacy is uncommon.

In many studies, experiencing little or no pain is found to be a very important contributing factor towards satisfaction with MA [6]. Although in our study pain was not found to be statistically significant, women who did not have more than expected pain reported satisfaction with MA. The selected proximate determinants, namely, the experiences of shorter duration and less amount of bleeding as well as shorter length of abortion process relate to high level of satisfaction with MA, controlling for other factors. As in other studies, many factors have been found to highly influence women’s satisfaction of MA.

The analysis of providers’ characteristics suggests that being doctor or a nurse or an ANM does not have a significant effect on women’s reported satisfaction with MA. However, women reported higher levels of satisfaction when the provider was older than 35 years. Also, women who reported no preference for a female provider were more likely to be satisfied than women who preferred a female provider. There is also some further indication that the sex of the provider played a role in women’s discomfort with the procedure; the study found that more women in the doctors’ group reported discomfort with the vaginal examination. In the context of Nepal, women generally feel uncomfortable having physical examination and especially vaginal examination conducted by male doctors. Therefore, greater discomfort with vaginal examination reported by women in the doctors’ group may be due to most doctors being men compared to nurse or ANM group who were all women.

The role of counseling on acceptance and satisfaction with MA has not been extensively studied. Almost all women in this study reported being counseled which may have played a role in the high level of satisfaction with MA service. An understanding of the steps of the procedure and signs of complications can significantly help women better address the common symptoms that are associated with MA. These counseling service proximate measures were nearly universal and similar by type of provider. Nearly all women responded “yes” to questions on whether the method or signs of complications were explained, contraceptive counseling provided, and the opportunity provided to ask questions. These variables were, therefore, not included for the multivariate analysis shown in Table 5. Perhaps because of high quality counseling on MA and anticipated complication by both type of providers, the level of satisfaction reported by women was high. The training of providers on MA placed high emphasis on counseling and communication, in addition to technical competence. Therefore, the findings from study hospital shown here may not hold broadly for Nepal. However, these findings suggest where counseling coverage and quality are higher, women are likely to be satisfied with the method.

Among the eight independent characteristics, only one – gestational age – was significantly related to the reported level of satisfaction. The odds ratio of high satisfaction was 1.43 for women with 8–9 weeks of pregnancy as compared to those with 5–7 weeks (Table 5). This is in contrast to the finding from Finland where shorter gestational age was associated with higher satisfaction and acceptability of medical abortion [22]. In the context of Nepal, access to safe abortion services was still limited at the time of the study (2009), especially in hilly and lowland regions. Women with 8–9 weeks of pregnancy may be concerned about terminating pregnancy safely or may have anticipated undergoing surgical abortion. The availability of MA may have therefore augmented their satisfaction. However, with the limited data, we are unable to test these hypotheses or explain reasons for the relatively higher satisfaction among women with longer first-trimester gestational age.

Providers’ age was significant, but not the length of experience in providing abortion. We also probed if preference for the sex of provider had any bearings on the reported satisfaction with MA. Among 1027 women included in the study, 366 (35.6%) had no preference, 657 (64%) preferred a female provider and the remaining four women preferred a male provider. Given little (0.4%) preference for male provider, we could only compare women with ‘no preference’ to those with a preference for a female provider and find that the former were more likely to report higher satisfaction than the latter. We were unable to extend the analysis further to examine if the concordance between the preference for sex of provider and of the actual provider resulted in greater reported satisfaction because the study did not document the sex of actual MA provider for each woman obtaining service. However, we know that doctors were mostly men (79%) and nurses and ANMs were all women. The study finds no difference in the level of satisfaction by provider type and thus indirectly confirms that the sex of the actual provider did not influence the reported satisfaction of MA.

Assessing satisfaction with MA was a secondary objective of the trial primarily designed to examine the safety and efficacy of MA provided by trained nurses and ANMs as compared to doctors. We were therefore, constrained by the limited number of questions included in the study on satisfaction. User satisfaction and client perspectives on methods and services can be obtained by objective observation, exit interviews with clients, and by experience of care [17, 20]. Our analysis is based on the subjective assessment of women of their abortion experience and of overall satisfaction with the method. Further research is needed to better define the objective measures of satisfaction and the drivers of such satisfaction.

From the data collected in this study, we find that satisfaction with MA provided by nurse and ANMs is as high as services provided by doctors. We also find that counseling on the method, sign of complications and on post-abortion contraception was nearly universal which may have contributed to high level of reported satisfaction. Therefore, medical abortion services provided by nurses or ANMs with appropriate training and counseling can be made available to all women in Nepal seeking early first trimester abortion.

## Conclusions

Women in Nepal seeking medical abortions in early first trimester of pregnancy express equal satisfaction with the service whether provided by trained nurses and ANMs or by doctors. In our study, practically all women were satisfied with the procedure and service irrespective of type of provider. Taken together with the findings of safety [1], this study supports extending safe and accessible MA services by trained nurses and ANMs to women seeking early first trimester pregnancy termination. These results have important implications for expanding access to safe abortion in resource-poor settings and in rural areas where doctors are in short supply and where trained nurse and ANMs can provide as safe, effective and highly satisfactory service to women as doctors. The study also highlights the importance of good quality counseling that contributes to satisfaction with MA.

## Abbreviations

ANM: Auxiliary nurse-midwife
CI: Confidence interval
MA: Medical abortion
OR: Odds ratio
SD: Standard deviation
SPSS: Statistical Package for Social Sciences

## References

[1] Warriner IK, Wang D, Huong NT, Thapa K, Tamang A, Shah I, Baird D, Meirik O (2011). Can midlevel health-care providers administer early medical abortion as safely and effectively as doctors? A randomized controlled equivalence trial in Nepal. Lancet.

[2] Abortion M (2011). Expanding access to safe abortion and saving women's lives: medical abortion. An international forum on policies, Programmes and services, 17−20 October 2004.

[3] Elul B, Ellertson C, Winikoff B, Coyaji K (1999). Side effects of mifepristone-misoporostol abortion versus surgical abortion – data from a trial in China, Cuba, and India. Contraception.

[4] Ngoc NTN (1999). Safety, efficacy and acceptability of mifepristone-misoporostol medical abortion in Vietnam. Int Fam Plan Perspect.

[5] Tran NT (2010). Feasibility, efficacy, safety and acceptability of mifepristone-misoprostol medical abortion in the democratic People’s Republic of Korea. Int J Gynaecol Obstet.

[6] World Health Organization (2012). Safe abortion: technical and policy guidance for health systems.

[7] Tamang A, Tuladhar S, Tamang J, Ganatra B, Dulal B (2012). Factors associated with choice of medical or surgical abortion among women in Nepal. Int J Gynecol Obstet.

[8] Olavarrieta CD, Ganatra B, Sorhaindo A, Karver TS, Seuc A (2015). Nurses versus physician-provision of early medical abortion in Mexico: a randomized controlled non-inferiority trial. Bull World Health Organ.

[9] Bruce J (1990). Fundamental elements of the quality of care: a simple framework. Stud Fam Plan.

[10] Romer T, Linsberger D (2009). User satisfaction with a levonorgestrel-releasing intrauterine system (LNG-IUS): data from an international survey. Eur J Contracept Reprod Health Care.

[11] Tewari R, Kay VJ (2006). Compliance and user satisfaction with the intra-uterine contraceptive device in family planning service: the results of a survey in fife, Scotland, august 2004. Eur J Contracept Reprod Health Care.

[12] Williams T, Schutt-Aine J, Cuca Y (2000). Measuring family planning service quality through client satisfaction exit interviews. Int Fam Plan Perspect.

[13] Blanc AK, Curtis SL, Croft TN (2002). Monitoring contraceptive continuation: links to fertility outcomes and quality of care. Stud Fam Plan.

[14] Bessinger RE, Bertrand JT (2001). Monitoring quality of care in family planning programs: a comparison of observations and client exit interviews. Int Fam Plan Perspect.

[15] Whittaker M (1996). Evaluating rural Bangladeshi women’s perspectives of quality in family planning services. Health Care Women International.

[16] Nakhaee N, Mirahmadizadeh AR (2005). Iranian women’s perceptions of family planning service quality: a client-satisfaction survey. Eur J Contracept Reprod Health Care.

[17] Measuring TK, Quality of Care: A Review of Previously Used Methodologies and Indicators (2016). Working paper two of the measuring and monitoring quality of services and quality of care project.

[18] Winikoff B (1995). Acceptability of medical abortion in early pregnancy. Fam Plan Perspect.

[19] World Health Organization (2017). Quality of care in contraceptive information and services, based on human rights standards: A checklist for health care providers.

[20] Salisbury C, Wallace M, Montgomery AA (2010). Patients’ experience and satisfaction in primary care: secondary analysis using multilevel modelling. BMJ.

[21] Bongaarts JA (1978). Framework for analyzing the proximate determinants of fertility. Popul Dev Rev.

[22] Honkanen H, von Hertzen H (2002). Users’ perspectives on medical abortion in Finland. Contraception.

